# Dopaminergic and behavioural changes in a loss‐of‐imprinting model of Cdkn1c

**DOI:** 10.1111/gbb.12422

**Published:** 2017-09-15

**Authors:** G. I. McNamara, B. A. Davis, M. Browne, T. Humby, J. W. Dalley, J. Xia, R. M. John, A. R. Isles

**Affiliations:** ^1^ Behavioural Genetics Group, MRC Centre for Neuropsychiatric Genetics and Genomics Neuroscience and Mental Health Research Institute, Cardiff University Cardiff UK; ^2^ School of Biosciences Cardiff University Cardiff UK; ^3^ School of Psychology Cardiff University Cardiff UK; ^4^ Department of Psychology University of Cambridge Cambridge UK; ^5^ Department of Psychiatry University of Cambridge Cambridge UK

**Keywords:** Dopamine, epigenetics, imprinted genes, p57kip2, reward, social dominance

## Abstract

The imprinted gene Cdkn1c is expressed exclusively from the maternally inherited allele as a consequences of epigenetic regulation. Cdkn1c exemplifies many of the functional characteristics of imprinted genes, playing a role in foetal growth and placental development. However, Cdkn1c also plays an important role in the brain, being key to the appropriate proliferation and differentiation of midbrain dopaminergic neurons. Using a transgenic model (Cdkn1c ^BACx1^) with a twofold elevation in Cdkn1c expression that mimics loss‐of‐imprinting, we show that increased expression of Cdkn1c in the brain gives rise to neurobiological and behavioural changes indicative of a functionally altered dopaminergic system. Cdkn1c ^BACX1^ mice displayed altered expression of dopamine system‐related genes, increased tyrosine hydroxylase (Th) staining and increased tissue content of dopamine in the striatum. In addition, Cdkn1c ^BACx1^ animals were hypersensitive to amphetamine as showed by c‐fos expression in the nucleus accumbens. Cdkn1c ^BACX1^ mice had significant changes in behaviours that are dependent on the mesolimbic dopaminergic system. Specifically, increased motivation for palatable food stuffs, as indexed on a progressive ratio task. In addition, Cdkn1c ^BACX1^ mice displayed enhanced social dominance. These data show, for the first time, the consequence of elevated Cdkn1c expression on dopamine‐related behaviours highlighting the importance of correct dosage of this imprinted gene in the brain. This work has significant relevance for deepening our understanding of the epigenetic factors that can shape neurobiology and behaviour.

Imprinted genes represent a class of genes that are monoallelically expressed, allelic expression being dependant on the parent of origin, as a result of epigenetic events initiated in the germline (Ferguson‐Smith 2011). This class of genes were identified following on from the observation of the non‐equivalence of the parental genomes in androgenetic, gynogenetic and parthenogenetic murine embryos resulting in embryonic fatality (McGrath & Solter 1984; Surani *et al*. 1984). The function of these genes converge on key aspects of mammalian physiology including placental development and function (Tunster *et al*. 2013), metabolism (Smith *et al*. 2006) and behaviour (Davies *et al*. 2015). Furthermore, imprinted gene function is sensitive to dosage as exemplified by human imprinting disorders that arise from improper imprinted gene expression levels. These include Beckwith–Wiedemann syndrome, Silver–Russell syndrome (SRS) (Demars & Gicquel 2012), Prader–Willi and Angelman syndromes (Buiting 2010).

*Cyclin dependant kinase inhibitor 1c* (*Cdkn1c*) is a maternally expressed imprinted gene located in the *Kcnq1* imprinting locus on mouse distal chromosome 7/human chromosome 11p15. *Cdkn1c* (aka p57^Kip2^) is a member of the Cip/Kip family of cyclin‐dependant kinase inhibitors (CDKi) (Lee *et al*. 1995; Matsuoka *et al*. 1995). Expression in the brain peaks embryonically with restricted postnatal and adult expression (Furutachi *et al*. 2013; Furutachi *et al*. 2015; Westbury *et al*. 2001). In the developing nervous system *Cdkn1c* plays a role in neurogenesis, migration and morphology (Itoh *et al*. 2007; Joseph *et al*. 2003; Tury *et al*. 2011; Ye *et al*. 2009). Independent of its CDKi function, *Cdkn1c* cooperates with *Nurr1*, to promote the proliferation and differentiation of midbrain dopaminergic neurons (Joseph *et al*. 2003). Maternally inherited loss‐of‐function of *Cdkn1c* results in reduced numbers of Nurr1‐positive and Th‐positive cells in the ventral midbrain on E18.5 (Joseph *et al*. 2003). Study of *Cdkn1c* function postnatally has been limited due to the perinatal lethality of a global knockout (Yan *et al*. 1997; Zhang *et al*. 1997). In the brain, experiments utilizing a conditional knockout of *Cdkn1c* have shown that this gene functions to maintain quiescence of adult neural stem cells (Furutachi *et al*. 2013; Furutachi *et al*. 2015).

We have previously showed a role for *Cdkn1c* in the behavioural symptoms associated with the imprinting disorder, SRS, specifically relating to hedonic responding to a palatable food stuff (McNamara *et al*. 2016). Here, we model the consequences of loss‐of‐imprinting of *Cdkn1c* using a BAC (bacterial artificial chromosome) transgenic mouse model (*Cdkn1c* ^BACx1^) that resulted in elevated *Cdkn1c* in a subset of tissues, including in the developing nervous system, at twofold the endogenous level (Andrews *et al*. 2007; John *et al*. 2001). We report that a double dosage of *Cdkn1c* results in an altered dopaminergic state in the mesolimbic system, with elevated Th and neurotransmitter levels in the striatum associated with increase Th‐positive cell number in the ventral tegmental area (VTA). Corresponding to this, changes in a number of behaviours, specifically motivation for a food reward and social dominance, were observed. These findings lend further support to the importance of genomic imprinting in mediating complex mammalian behaviours and emphasize the significance of *Cdkn1c* expression for dopaminergic system function.

## Materials and methods

### *Animals*

All procedures were conducted in accordance with the requirements of the UK Animals (Scientific Procedures) Act 1986, under the remit of Home Office licence number 30/2673 with additional ethical approval at Cardiff University.

The experimental line *Cdkn1c* ^BACx1^ possesses one copy of a bacterial artificial chromosome that spans the *Cdkn1c* gene and two other genes, *Phlda2* and *Slc22a18*. The reporter line *Cdkn1c* ^BACLacZ^ possesses a modified version of this BAC with a β*‐galactosidase* reporter construct inserted into the *Cdkn1c* locus, disrupting *Cdkn1c* expression (John *et al*. 2001). *Cdkn1c* ^BACLacZ^ functioned as a control for the experimental line to attribute any phenotypes observed specifically to the over expression of *Cdkn1c* (i.e. present in the line *Cdkn1c* ^BACx1^ and absent in the line *Cdkn1c* ^BACLacZ^). Both lines are maintained on a C57BL/6J background and have been bred onto this background for >8 generations. For embryonic tissue wild‐type (WT) C57BL/6J females aged 6–8 weeks were paired with a *Cdkn1c* ^BACx1^ male. Presence of a vaginal‐plug designated embryonic day (E)0.5. On E18.5, embryos were isolated and forebrain was separated from mid‐ and hindbrain. Tissue was snap frozen and stored at −80°C until processing.

Subjects were male mice. For the social dominance tube test task experiment *n* = 71 animals were used in total: *Cdkn1c* ^BACx1^ (*n* = 23) and their WT littermates (*n* = 19); *Cdkn1c* ^BACLacZ^ (*n* = 14) and their WT littermates (*n* = 15). A subset of these was used for progressive ratio testing: *Cdkn1c* ^BACx1^ (*n* = 14) and their WT littermates (*n* = 13); *Cdkn1c* ^BACLacZ^ (*n* = 17) and their WT littermates (*n* = 9). Animals were between 8 and 12 weeks old for social dominance testing and 13 and 15 weeks at the start of progressive ratio testing. Standard laboratory chow was available *ad libitum*, but just prior to and during the progressive ratio experiment, water was restricted to 2 h access per day. This regime maintained the subjects at ≈90% of free‐feeding body weight. All behavioural testing was performed in the light phase (lights on 0700 h, for 12‐h).

### *RNA extraction and qPCR*

Tissue for RNA analysis was obtained following cervical dislocation and isolation of the brain. To assess sensitivity to amphetamine an intraperitoneal injection of saline or 0.5 mg/kg D‐amphetamine (Tocris Bioscience, Bristol, UK) was administered. Brains were isolated 60 min following injection by cervical dislocation and ventral striatum was isolated. Embryonic RNA was extracted using PARIS kit (Life Technologies, UK). Adult RNA was extracted using standard phenol extraction methods and DNase treated using RNAse‐free DNase treatment and removal kit (Applied Biosystems, UK) according to the manufacturer's instructions. Expression was normalized (ΔCt) to the geometric mean Ct value of *βactin* and *Hprt* as these genes have previously been verified in‐house. Statistical analysis was carried out on transformed ΔCt values.

### *Immunohistochemistry and image analysis*

Immunohistochemistry was carried out as previously described (McNamara *et al*. 2016). Briefly, animals were transcardially perfused with 10% formalin fixative (Sigma‐Adrich, UK) and brains were removed. Following post‐fixing overnight, brains were removed to a solution of phosphate‐buffered saline containing 30% sucrose and 40 µm sections were obtained on a freezing microtome. Immunostaining was carried out on free‐floating sections. For striatal analysis, adjacent sections were incubated in a single primary antibody [anti‐NeuN (1:1000) (Abcam, UK) or rabbit polyclonal anti‐Th (1:1000) (Abcam)] in 3% NGS TBS‐T overnight at 4°C. For analysis of the VTA, a separate cohort of tissue was prepared as above and staining was carried out on every 1 in 6 sections. The VECTASTAIN ABC kit (Vector Labs, Peterborough, UK) and diaminobenzidine tetrahydrochloride peroxidise substrate kit (Vector Labs, UK) were used as per manufacturer's instructions. Images were acquired at × 1.5 (for Th immunoreactivity) or × 5 (for NeuN immunoreactivity) magnification using Leica Olympus DP73. The × 20 Th cell counts in VTA and substania nigra *pars compacta* (SNc) were acquired using Olympus BX41 brightfield microscope with slide‐scanning adapter. For Th immunoreactivity all sections containing the striatum were imaged. For NeuN 10 sections distributed evenly throughout the striatum were chosen for analysis. A single image was taken of the striatum and another of the adjacent cortex, for each hemisphere, a total of 20 images per animal. All image analysis was carried out using ImageJ. For striatal Th average optical density of the striatum, separated into dorsal and ventral regions, was obtained and the average background staining from the cortex was subtracted to give a value comparable between sections. Cell number (NeuN and Th) was calculated using ImageJ cell counter plugin (NIH ImageJ software version 1.45s; http://rsb.Info.nih.gov/ij/) on the 8‐bit black and white images. Th cell number in VTA and SNc was counted manually by experimenter blind to genotype.

### *Whole‐tissue neurochemistry*

Animals were killed by exposure to a rising concentration of carbon dioxide and cervical dislocation. The brains were rapidly removed and the dorsal striatum dissected. Tissue aliquots were derived from both hemispheres and homogenized in 200 µl of 0.2 m perchloric acid by an ultrasonic cell disruptor (Microson, UK). Levels of NA, DA and 5‐HT were determined in the supernatant by reversed‐phase, high‐performance liquid chromatography (HPLC), as described previously (Dalley *et al*. 2002).

### *Progressive ratio*

For the duration of testing animals were on a restricted water access schedule, water provided for 2 h immediately after testing. Briefly, testing took place in a nine‐hole box modified for use in mice. During testing a nose‐poke (NP) in the illuminated hole resulted in the presentation of 20 µl of an 8% sucrose reinforce. Collection of this reward initiated a subsequent trial. Continuous reinforcement (CRF – i.e. one NP required for reward delivery) was carried out for 4 days. Following this, a progressive‐ratio schedule was carried out. Number of NPs required ascended linearly every four trials (Ratio 4, FR4) for three sessions, followed every two trials (Ratio 2, FR2) a further three sessions. The maximum number of NP an animal was willing to make to receive a reward is deemed break‐point (BP), and is an indication of the animal's motivation to work for a reward. These progressive‐ratio sessions were followed by four more CRF sessions.

Following this basal analysis, single probe trials at the ratio FR2 progressive‐ratio schedule were carried out using 2% (w/w) sucrose and calorie‐free saccharin [0.1% (w/w)]. To exclude effects of neophobia on saccharin BP, animals were trained to obtain a reward of 0.1% saccharin in a CRF schedule for 2 days prior to this a probe trial at FR2.

Two infrared beams at front and rear of arena provided a measure of activity during trials. Each break in the beam was counted and total beam breaks in a trial was analysed.

### *Social dominance test*

The tube test was carried out as described in Garfield *et al*. 2011. Briefly, the test apparatus consisted of a 30 cm, transparent tube with a 3.5 cm diameter placed in an opaque arena to obscure view of the environment. Testing was carried out in dimmed light conditions. At the beginning of each trial two animals were introduced into the tube from both ends and released simultaneously. A trial was complete when one animal fully backed out of the tube. The animal that did not back out was considered the dominant animal of the trial. Each dyadic encounter consisted of one transgenic and one WT animal, these did not differ in weight by more than 10%. Each transgenic animal had three encounters with three different, unfamiliar, WT animals. The number of encounters won by either transgenic or WT animals was recorded.

### *Statistical analysis*

All statistical analysis was carried out using SPSS 20.0 (SPSS, USA). For analysis of *Dat* expression, dopamine concentration in the dorsal striatum and Th cell number in VTA and SNc, Student's *t*‐tests were carried out with GENOTYPE (WT or transgenic – either *Cdkn1c* ^BACx1^ or *Cdkn1c* ^BACLacZ^) as a grouping variable. For *Th* expression and IHC analysis a repeated measure anova (analysis of variance) was carried out with GENOTYPE as a between subject factor and REGION as a within subject factor. For analysis of progressive ratio a series of mixed anovas were carried out on the data with a between subject factor of GENOTYPE and within subject factors of SESSION or SOLUTION (sucrose or saccharin) where appropriate. Bonferroni *post‐hoc* tests were carried out where appropriate. Activity during progressive‐ratio testing was analysed using a Student's *t*‐tests were carried out with GENOTYPE as a grouping variable. Dominance behaviours in the tube test were assessed by a binomial non‐parametric test where all encounters were coded ‘0’ for those won by WT or coded ‘1’ for those won by transgenic (either *Cdkn1c* ^BACx1^ or *Cdkn1c* ^BACLacZ^). Expected proportion was 0.5 assuming each encounter was equally likely to be won by either WT or transgenic.

## Results

### *Increased Cdkn1c dosage results in a modified basal dopaminergic system in the striatum*

**Figure 1:**
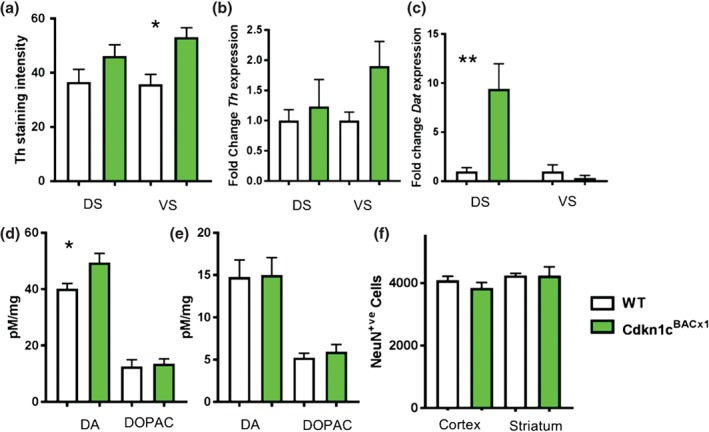
**Elevated expression of Cdkn1c results in an altered dopaminergic state in the striatum in Cdkn1c** ^**BACx1**^ **animals.** (a) Th immuno‐reactivity in the striatum (b) Adult Th expression normalized to WT (c) Dat expression normalized to WT. Whole tissue dopamine concentration in DS (d) and VS (e). Average NeuN^+ve^ cell count in striatum and cortex for Cdkn1c ^BACx1^. Dorsal striatum (DS) ventral striatum (VS) Data shown ±SEM. *P < 0.05, **P < 0.01.

As previously showed (Andrews *et al*. 2007; John *et al*. 2001), *Cdkn1c* expression was elevated in the forebrain in *Cdkn1c* ^BACx1^ animals on embryonic day (E)18.5 [*t*(5) = −6.9, *P* = 0.001] (Fig. S1 in Appendix S1, Supporting Information). In adult *Cdkn1c* ^BACx1^ animals, elevated expression of *Cdkn1c* resulted in an increase in Th protein immunoreactivity in the ventral striatum [*t*(6) = −3.49, *P* = 0.013] but not the dorsal striatum [*t*(6) = −1.5, *P* = 0.18] of *Cdkn1c* ^BACx1^ animals (Fig. 1a). Correspondingly, *Th* mRNA was increased 1.9‐fold in the ventral striatum, although this did not reach statistical significance [*t*(6) = −1.89, *P* = 0.11]. Th mRNA was unchanged in the dorsal striatum [*t*(6) = −0.76, *P* = 0.48] (Fig. 1b). There was no significant change in the *Dopamine transporter* (*Dat*) mRNA in the ventral striatum [*t*(5) = 1.5, *P* = 0.19] of *Cdkn1c* ^BACx1^ animals compared with WT littermates. However, in the dorsal striatum there was a ninefold increase in *Dat* mRNA [Fig. 1c; *t*(5) = 4.696, *P* = 0.005] in the dorsal striatum, this was accompanied by a 20% increase in whole tissue level of dopamine in the dorsal striatum of *Cdkn1c* ^BACx1^ animals [Fig. 1d; *t*(14) = −2.298, *P* = 0.038], without a change in the metabolite DOPAC [*t*(14) = −0.323, *P* = 0.75] or in turnover [*t*(14) = 0.874, *P* = 0.397, data not shown]. Despite the elevated Th protein staining in the ventral striatum, there was no effect of elevated *Cdkn1c* expression on dopamine, DOPAC or turnover in this region [dopamine: *t*(14) = −0.8, *P* = 0.93; DOPAC: *t*(14) = −0.63, *P* = 0.54; turnover: *t*(14) = 0.25, *P* = 0.81] (Fig. 1e). Given the cell‐cycle regulatory function of *Cdkn1c*, an important histological feature to assess was total neuronal cell number. There was no effect of elevated *Cdkn1c* on striatal [*t*(8) = 0.23, *P* = 0.98] or surrounding cortical cell number [*t*(8) = 1.15, *P* = 0.28] as determined by number of NeuN positive cells (Fig. 1f).

In all measures, the *Cdkn1c* ^BACLacZ^ transgenic control mice showed no phenotypic differences from WT (see Fig. S2a–e in Appendix S1).

### Cdkn1c^*BACx1*^ *mice have an altered neural response to amphetamine*

**Figure 2:**
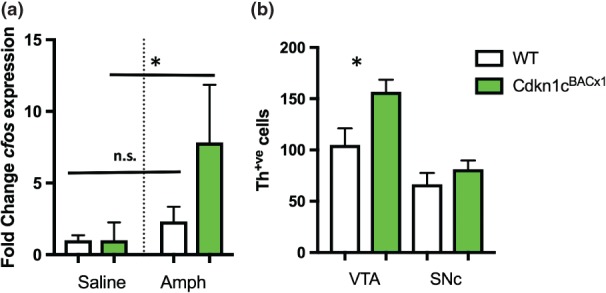
**Cdkn1c** ^**BACx1**^ **animals display increased neural reactivity to an intraperitoneal injection of amphetamine associated with increased mesolimbic input cell number.** Cfos expression in ventral striatum following a saline of amphetamine was significantly increased only in Cdkn1c ^BACx1^ animals. Data shown is mean fold change ± SEM. *P < 0.05 main effect of DRUG. Cdkn1c ^BACx1^ had significantly more Th‐positive cells than WT in VTA, but not SNc. Data shown is mean cell number per section ± SEM. *P < 0.05.

The ventral striatum encompasses the nucleus accumbens, a structure central to reward processing, and receives a direct dopaminergic input from the VTA. To probe the altered dopaminergic phenotype of *Cdkn1c* ^BACx1^ animals further we assessed the neural responsivity of this region to a single dose of amphetamine by measuring transcript levels of the immediate early gene *cfos* 60‐min post‐injection. Following a low dose (0.5 mg/kg) injection of amphetamine anova showed a GENOTYPE*DRUG interaction (*F*_1,17_ = 8.687, *P* = 0.009), with *post‐hoc* analysis showing increased *cfos* expression in the ventral striatum in animals with elevated *Cdkn1c* (Fig. 2a; *F*_1,17_ = 7.467, *P* = 0.014) but not in their WT littermates (*F*_1,17_ = 1.827, *P* = 0.194). This finding implies a greater response magnitude in cells of *Cdkn1c* ^BACx1^ animals or a larger number of cells activated in response to amphetamine stimulation. To address this, Th‐positive cells in the VTA, the mesolimbic input to the ventral striatum, were quantified. *Cdkn1c* ^BACx1^ had significantly more Th‐positive cells in the VTA than WT [Fig. 2b; *t*(8) = 2.32, *P* = 0.049]. There was no difference in Th‐positive cell number in the adjacent SNc [Fig. 2b; *t*(8) = 0.98, *P* = 0.37].

### *Elevated* Cdkn1c *dosage increases breakpoint on a progressive‐ratio task*

**Figure 3:**
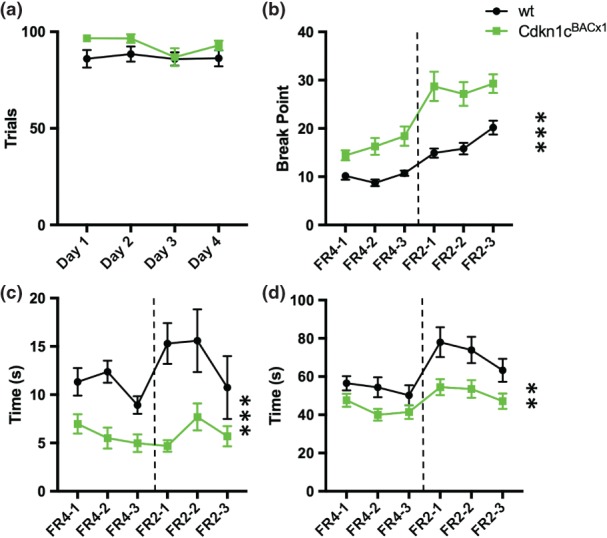
**Cdkn1c** ^**BACx1**^ **animals show increased motivation to obtain a sucrose reward compared with WT.** (a) Average number of trials completed during CRF trials. When minimal work (i.e. a single nose poke) was required to obtain a reward there was no difference in number of trails completed between Cdkn1c ^BACx1^ animals and their WT littermates. (b) When the number of nose‐pokes required to obtain a reward increased within a session, Cdkn1c ^BACx1^ made more nose‐pokes that WT before stopping (BP). (c) Duration of time between successive nose‐pokes was shorter in Cdkn1c ^BACx1^ animals. Average inter nose‐pokes interval. (d) Consequently, average time to complete a trial was shorter in Cdkn1c ^BACx1^ animals. Data shown ±SEM. **P < 0.01, ***P < 0.001.

During CRF trials, where a single NP elicited an 8% sucrose reward, there was no difference in number of trials completed between WT and *Cdkn1c* ^BACx1^ animals [Fig. 3a; main effect of GENOTYPE: *F*_1,23_ = 2.51, *P* = 0.127]. However, in a progressive‐ratio schedule, during which the number of NPs required to receive a reward ascends within a session, *Cdkn1c* ^BACx1^ animals had a 1.5‐ to 2‐fold higher BP than their WT littermates [Fig. 3b; *F*_1,23_ = 17.109, *P* < 0.001). This implies that *Cdkn1c* ^BACx1^ will work harder to receive a reward than WT. In addition to a higher BP, *Cdkn1c* ^BACx1^ animals had a shorter inter‐NP interval [Fig. 3c; *F*_1,23_ = 28.56, *P* < 0.001), and were quicker to complete trials [Fig. 3d; *F*_1,23_ = 12.442, *P* = 0.002). Importantly, there was no general difference in activity as measured by total beam breaks during the task (*F*_1,23_ = 0.179, *P* = 0.676) (data not shown). Together, these data indicate that twofold‐elevated expression of *Cdkn1c* increases directedness as well as motivation to obtain a sucrose reward.

In all these measures on the progressive ratio task, the *Cdkn1c* ^BACLacZ^ transgenic control mice showed no phenotypic differences from WT (Fig. S3a–d, Appendix S1).

### *Performance of* Cdkn1c^*BACx1*^ *mice is driven by motivation to obtain reward*

**Figure 4:**
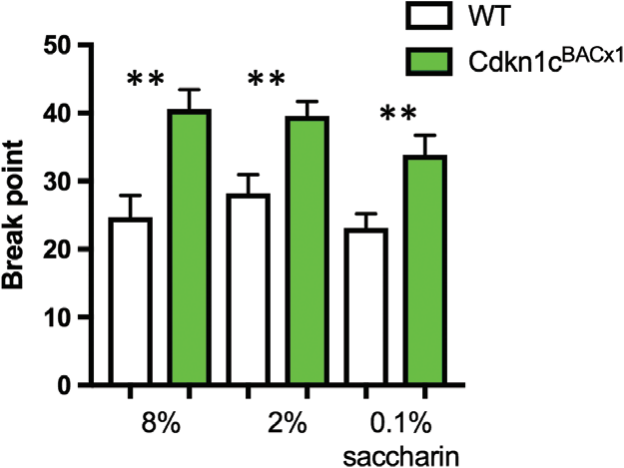
**Cdkn1c** ^**BACx1**^ **maintained elevated BP when presented with a less palatable sucrose concentration or a calorie‐free sweetener**. Cdkn1c ^BACx1^ animals maintained a higher average BP compared with WT when sucrose concentration was reduced from 8% to 2%. Cdkn1c ^BACx1^ animals maintained a higher BP in an FR2 schedule than WT animals when working for the calorie‐free sweetener, saccharin. Data shown ±SEM. **P < 0.01.

We explored the motivational phenotype of *Cdkn1c* ^BACx1^ mice on the progressive ratio task further by assessing the effect of manipulations of the reinforcer reward at a FR2 schedule. Firstly, we decreased the sucrose solution from 8% to 2%. Animals had equivalent breakpoints for 2% and 8% sucrose [Fig. 4; *t*(24) = 0.585, *P* = 0.56], and animals with elevated *Cdkn1c* expression continued to work harder than WT littermates to receive a 2% sucrose reward, as indexed by a significantly higher BP [*t*(23) = −3.334, *P* = 0.003].

To differentiate between the calorific and hedonic rewarding properties of sucrose, the animals' motivation to work calorie‐free saccharin [0.1% (w/w) saccharin] was assessed in the progressive ratio schedule. Animals were first trained to consume saccharin on a CRF schedule for 2 days with *Cdkn1c* ^BACx1^ and WT animals completing an average of 67.5 (5.2 SEM) and 76.14 (4.6 SEM) trials, respectively. Animals were then moved onto an FR2 schedule. All animals were less motivated to work for saccharin compared with 8% sucrose, as indexed by a decrease in BP [Fig. 4; main effect of SOLUTION (*F*_1,23_) = 10.293, *P* = 0.004]. Bonferroni *post hoc* test showed this to be driven primarily by the significant decrease in BP of *Cdkn1c* ^BACx1^ mice (*F*_1,23_ = 10.578, *P* = 0.004) compared with a non‐significant decrease in WT littermates (*F*_1,23_ = 0.174, *P* = 0.174). Nevertheless, despite this overall decrease, BP remained significantly higher in *Cdkn1c* ^BACx1^ mice than WT even when consuming saccharin [Fig. 4; *t*(23) = −2.872, *P* = 0.009].

### *Elevated* Cdkn1c *dosage increases social dominance*

**Figure 5:**
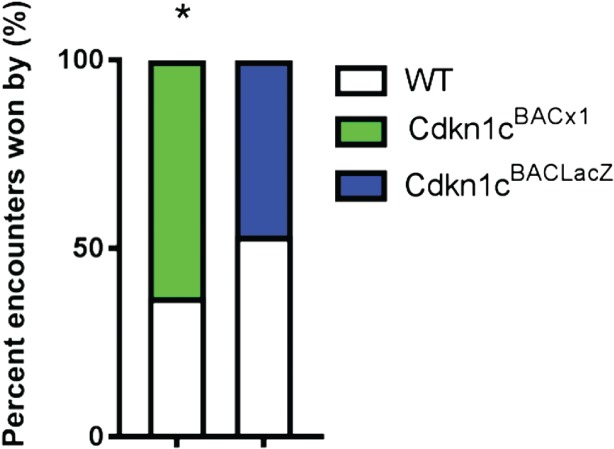
**Cdkn1c** ^**BACx1**^ **males are more dominant in a tube test.** (a) Cdkn1c ^BACx1^ animals won significantly more encounters against unfamiliar animals in the tubes test than WT. There was no difference in proportion of encounters won between Cdkn1c ^BACLacZ^ and WT animals.

Successful performance in a tube test is associated with a dominant status in rodents (Lindzey *et al*. 1961). When *Cdkn1c* ^BACx1^ males were paired with unfamiliar, weight‐matched WT males, the transgenic animals won significantly more encounters (Fig. 5; 63.1% vs. 36.9%, *χ* ^2^ = 4.45, *P* = 0.046). There was no significant difference in the proportion of encounters won by the control *Cdkn1c* ^BACLacZ^ males compared with unfamiliar, weight‐matched WT animals (Fig. 5; 46.7% vs. 53.3%, *χ* ^2^ = 0.2, *P* = 0.655).

## Discussion

In this study, we examined the consequence on brain and behaviour of an increase in expression of *Cdkn1c*, a maternally expressed imprinted gene critical for early development. We show that a twofold‐elevated expression of *Cdkn1c* alone, at levels that model loss‐of‐imprinting, induces a physiologically and behaviourally altered dopaminergic state. Specifically, elevated *Cdkn1c* expression caused increased striatal *Th* and *Dat*, elevated Th immunoreactivity and regional‐specific increases in dopamine. The *Cdkn1c* ^BACx1^ animals also showed an increased *cfos* reactivity to a stimulant in the ventral striatum, increased motivation to obtain a palatable reward, and increased social dominance. Although we can not draw conclusive causal relationships between elevated *Cdkn1c*, altered neurobiology and the observed behavioural phenotypes, this work underlines the importance of correct imprinted gene expression for brain and behaviour. Additionally, as the expression of rodent *Cdkn1c* is sensitive to maternal diet (Van de Pette *et al*. 2017; Vucetic *et al*. 2010b) and postnatal care (Pena *et al*. 2014), dysregulation of this gene may underpin some aspects of the neural and behavioural phenotypic consequences associated these adverse early life environments.

In the dorsal striatum, animals over expressing *Cdkn1c* were found to have an increased tissue level of dopamine, a finding emphasized by a significant increase in *Dat* mRNA levels compared with WT littermates and an increase in *Th* mRNA and protein expression in the ventral striatum. The increase in dopamine levels and the reuptake transporter mRNA, in absence of an increase in the metabolite DOPAC, occurs without a significant change in Th mRNA or protein. The HPLC analysis on whole postmortum tissue is not reflective of neurotransmitter dynamics *in vivo* and further studies using *in vivo* microdialysis may be more informative in future (Usiello *et al*. 2000). A possible underlying source for the increase in Th in the ventral striatum is an increase in the input cell number from the VTA. This convergent evidence highlights the altered basal dopaminergic state of animals overexpressing *Cdkn1c*. Given that *Cdkn1c* is a cell‐cycle regulator a change, in addition to the observation of an increase in Th‐positive cells in the VTA, an increase in total cell number may underlie these changes. However, this did not appear be the case as total neuronal cell number was not different between *Cdkn1c* ^BACx1^ animals and their WT littermates in either the striatum or surrounding cortex. Moreover, there was no increase in Th‐positive cells in the VTA‐adjacent nucleus, the SNc. The dissociation between increased Th‐positive cells of the midbrain in the absence of a global increase in neuronal cells number is not unexpected, as the function of *Cdkn1c* in promotion of dopaminergic neuron proliferation is known to be independent of the CDKi domain (Joseph *et al*. 2003).

We show that a consequence of a physiologically relevant increase in *Cdkn1c* is a hypersensitivity to amphetamine, a drug of abuse in humans. This injection of amphetamine did not alter *cfos* expression in the WT littermates. This concentration of amphetamine lower than was used previously to increase locomotor activity (McNamara *et al*. 2016) and plausibly was too modest for an observable effect in WT animals using this analysis technique. The location of this neural hypersensitivity is significant as it occurred specifically in the ventral striatum, containing the nucleus accumbens. A number of immediate early genes, including *cfos,* are rapidly induced in the nucleus accumbens following exposure to an addictive substance (Hope *et al*. 1994), and this region, part of the brain's ‘reward circuitry’ (Russo & Nestler 2013; Smith *et al*. 2011; Wise 2013) is known to undergo significant changes that underlie the behavioural transition to addiction (Nestler 2001). The altered sensitivity of cells in this region to amphetamine emphasises the altered dopaminergic state as a consequence of elevated *Cdkn1c*.

Alterations in neural responsivity to injection of a rewarding substance were mirrored in differences in behavioural responding to a palatable food reward. *Cdkn1c* ^BACx1^ animals had an increased motivational drive to obtain a sucrose solution. In addition, the average inter‐NP interval and time to complete a trial was shorter in *Cdkn1c* ^BACx1^ animals. These latter measures relate to how rapidly an animal obtains successive rewards. These differences suggest that *Cdkn1c* ^BACx1^ animals spent more time engaging in activities relating to obtaining the sucrose reward, compared with WT. We have previously showed that *Cdkn1c* ^BACx1^ animals were hypoactive in an arena in comparison to their WT littermates (McNamara *et al*. 2016). However, activity levels do not appear to be a confounding factor in behaviour in this task as there was no effect of genotype on beam breaks, a measure of locomotor activity, during task performance. Bonferroni *post‐hoc* tests showed that the decrease in BP when working to obtain the calorie‐free saccharin in comparison to 8% sucrose was statistically significant only in *Cdkn1c* ^BACx1^ animals and not their WT littermates. This indicates that *Cdkn1c* ^BACx1^ performance was more influenced by the calorific reward of sucrose and less by the palatability. However, BP in *Cdkn1c* ^BACx1^ animals remained elevated with respect to WT littermates for saccharin, indicating a heightened motivational drive for both caloric and non‐caloric rewards. This finding is initially at odds with our previously published work indicating a blunted hedonic response to sucrose in animals with elevated *Cdkn1c* expression (McNamara *et al*. 2016). Intriguingly, dopamine release in dorsal and ventral striatum has recently been shown to be functionally distinct with respect to hedonic and nutritional value sensing (Tellez *et al*. 2016). The regional specificity of the changes in the dopaminergic system may reflect a differential input magnitude to the dorsal and ventral striatum as a consequence of elevated *Cdkn1c*, and may underlie the observed behavioural phenotype.

In addition, we also explored social dominance behaviour in the *Cdkn1c* ^BACx1^ animals. Previous work has shown that social dominance is governed in part by dopamine in rodents (Jupp *et al*. 2016) and that more dominant animals also show increased motivation for reward (Davis *et al*. 2009). However, the relationship between dopamine and social dominance is not clear‐cut, and other studies have implicated additional brain systems (Wang *et al*. 2011). Nevertheless, we particularly wanted to explore this behaviour here in light of our previous work with another imprinted gene, *Grb10*, where we showed that mice carrying a paternal knockout (*Grb10* ^*patKO*^) showed increased social dominance (Garfield *et al*. 2011). Mirroring the work with *Grb10* ^*patKO*^ mice, *Cdkn1c* ^BACx1^ mice were also more likely to win a tube‐test encounter with unfamiliar animals than their WT littermates. This increased success was absent in our additional control line, *Cdkn1c* ^BACLacZ^, again indicating that this behaviour was a consequence of elevated *Cdkn1c* expression alone. From this data set, we can not exclude the possibly that the hypoactivity we have previously reported in these animals influences performance in this task, rather than altered dominance *per se*. Additionally *Cdkn1c* expression peaks at E13.5 (Westbury *et al*. 2001), therefore, it is possible that these phenotypes are due to a general dysregulation early in development as a consequence of elevated expression from the BAC transgene, not limited to the dopaminergic system. It is possible to limit these effects to a limited subset of tissues where the BAC is expressed, including the developing nervous system (Andrews *et al*. 2007). Nonetheless, this is the first explicit demonstration of a convergent role for oppositely imprinted genes on a behavioural function in a directly comparable task, paralleling other functional studies indicating a convergent role for imprinted genes in placental function, energy homeostasis and thermogenesis (Cleaton *et al*. 2014).

Current data suggests that generally imprinted genes are relatively insensitive to environmental manipulations such as *in utero* nutritional programming and instances of dosage regulation by relaxation of imprinting are rare and likely to be highly regulated (Radford *et al*. 2012). However, one such exception is *Cdkn1c* where expression in brain is sensitive to adverse early life events. Specifically, both low maternal dietary protein (Vucetic *et al*. 2010b), and the degree of postnatal maternal care (Pena *et al*. 2014) lead to increased *Cdkn1c* expression in the brain. For the case of both low maternal dietary proteins, this appears to be due to de‐repression of the normally silent paternally inherited allele of *Cdkn1c* (Van de Pette *et al*. 2017). Both of these early life manipulations are associated with changes in the dopamine system and the response to reward (Pena *et al*. 2014; Vucetic *et al*. 2010b). Indeed the contribution of maternal diet (Ong & Muhlhausler 2011; Vucetic *et al*. 2010a), and maternal care (Hall *et al*. 1999), to the development and function of the offspring dopamine system is well established. Whilst it is likely that the expression of many genes is affected by these adverse early life events, our findings, where *Cdkn1c* expression alone is elevated through genetic modification, would suggest that changes in expression of this imprinted gene could play a key role in mediating the associated brain and behavioural changes. However, the extent to which increased *Cdkn1c* expression mediates phenotypic changes brought about by adverse early life events, remains to be clarified.

In conclusion, we have shown that elevated expression of *Cdkn1c*, equivalent to loss‐of‐imprinting, results in an altered dopaminergic neural state and increased motivation for reward and social dominance, the latter finding providing the first example of a convergent role for imprinted genes on a behavioural function. The behavioural consequences of an altered dopamine system are further supported by our previous findings using this model where we showed altered locomotor activity and sensory‐motor gating deficits (McNamara *et al*. 2016). This study reemphasises the function of imprinted genes on complex adult behaviour and, as *Cdkn1c* expression is sensitive to the early life environment (Pena *et al*. 2014; Van de Pette *et al*. 2017; Vucetic *et al*. 2010b), shows a potential role of the imprinted gene *Cdkn1c* in mediating the changes dopaminergic function seen following such adverse early life events. Finally, this work may have also have implications for the imprinting disorders Beckwith–Wiedemann, Silver–Russell and iMAGE syndromes that are associated with altered *CDKN1C* dosage (Eggermann *et al*. 2014).

## Supporting information


**Figure S1:** Cdkn1c ^BACx1^ animals had elevated neural Cdkn1c expression at e18.5 as determined by qPCR. Data shown ±SEM. **P < 0.01.
**Figure S2:** There was no effect of genotype on dopaminergic state in the striatum of Cdkn1c ^BACLacZ^ animals and WT littermates. (a) Adult Th expression normalized to WT (b) Dat expression normalized to WT. (c) Whole tissue dopamine concentration. (d) Th immuno‐reactivity in the striatum (e). Average NeuN^+ve^ cell count. Dorsal striatum (DS) ventral striatum (VS) Data shown ±SEM.
**Figure S3:** There was no effect of genotype on motivation to obtain a sucrose reward compared between Cdkn1c ^BACLacZ^ animals and WT littermates. (a) Average number of trials completed during CRF trials (b) BP when the number of nose‐pokes required to obtain a reward increased within a session. (c) Duration of time between successive nose‐pokes (d) Average time to complete a trial. Data shown ±SEM.

## References

[gbb12422-bib-0001] Andrews, S.C. , Wood, M.D. , Tunster, S.J. , Barton, S.C. , Surani, M.A. & John, R.M. (2007) Cdkn1c (p57Kip2) is the major regulator of embryonic growth within its imprinted domain on mouse distal chromosome 7. BMC Dev Biol 7, 53. 17517131 10.1186/1471-213X-7-53 PMC1891291

[gbb12422-bib-0002] Buiting, K. (2010) Prader‐Willi syndrome and Angelman syndrome. Am J Med Genet C Semin Med Genet 154C, 365–376. 20803659 10.1002/ajmg.c.30273

[gbb12422-bib-0003] Cleaton, M.A. , Edwards, C.A. & Ferguson‐Smith, A.C. (2014) Phenotypic outcomes of imprinted gene models in mice: elucidation of pre‐ and postnatal functions of imprinted genes. Annu Rev Genomics Hum Genet 15, 93–126. 24898037 10.1146/annurev-genom-091212-153441

[gbb12422-bib-0004] Dalley, J.W. , Theobald, D.E. , Eagle, D.M. , Passetti, F. & Robbins, T.W. (2002) Deficits in impulse control associated with tonically‐elevated serotonergic function in rat prefrontal cortex. Neuropsychopharmacology 26, 716–728. 12007742 10.1016/S0893-133X(01)00412-2

[gbb12422-bib-0005] Davies, J.R. , Dent, C.L. , McNamara, G.I. & Isles, A.R. (2015) Behavioural effects of imprinted genes. Curr Opin Behav Sci 2, 28–33.

[gbb12422-bib-0006] Davis, J.F. , Krause, E.G. , Melhorn, S.J. , Sakai, R.R. & Benoit, S.C. (2009) Dominant rats are natural risk takers and display increased motivation for food reward. Neuroscience 162, 23–30. 19393296 10.1016/j.neuroscience.2009.04.039

[gbb12422-bib-0007] Demars, J. & Gicquel, C. (2012) Epigenetic and genetic disturbance of the imprinted 11p15 region in Beckwith‐Wiedemann and silver‐Russell syndromes. Clin Genet 81, 350–361. 22150955 10.1111/j.1399-0004.2011.01822.x

[gbb12422-bib-0008] Eggermann, T. , Binder, G. , Brioude, F. , Maher, E.R. , Lapunzina, P. , Cubellis, M.V. , Bergada, I. , Prawitt, D. & Begemann, M. (2014) CDKN1C mutations: two sides of the same coin. Trends Mol Med 20, 614–622. 25262539 10.1016/j.molmed.2014.09.001

[gbb12422-bib-0009] Ferguson‐Smith, A.C. (2011) Genomic imprinting: the emergence of an epigenetic paradigm. Nat Rev Genet 12, 565–575. 21765458 10.1038/nrg3032

[gbb12422-bib-0010] Furutachi, S. , Matsumoto, A. , Nakayama, K.I. & Gotoh, Y. (2013) p57 controls adult neural stem cell quiescence and modulates the pace of lifelong neurogenesis. EMBO J 32, 970–981. 23481253 10.1038/emboj.2013.50 PMC3616292

[gbb12422-bib-0011] Furutachi, S. , Miya, H. , Watanabe, T. , Kawai, H. , Yamasaki, N. , Harada, Y. , Imayoshi, I. , Nelson, M. , Nakayama, K.I. , Hirabayashi, Y. & Gotoh, Y. (2015) Slowly dividing neural progenitors are an embryonic origin of adult neural stem cells. Nat Neurosci 18, 657–665. 25821910 10.1038/nn.3989

[gbb12422-bib-0012] Garfield, A.S. , Cowley, M. , Smith, F.M. , Moorwood, K. , Stewart‐Cox, J.E. , Gilroy, K. , Baker, S. , Xia, J. , Dalley, J.W. , Hurst, L.D. , Wilkinson, L.S. , Isles, A.R. & Ward, A. (2011) Distinct physiological and behavioural functions for parental alleles of imprinted Grb10. Nature 469, 534–538. 21270893 10.1038/nature09651 PMC3031026

[gbb12422-bib-0013] Hall, F.S. , Wilkinson, L.S. , Humby, T. & Robbins, T.W. (1999) Maternal deprivation of neonatal rats produces enduring changes in dopamine function. Synapse 32, 37–43. 10188636 10.1002/(SICI)1098-2396(199904)32:1<37::AID-SYN5>3.0.CO;2-4

[gbb12422-bib-0014] Hope, B.T. , Nye, H.E. , Kelz, M.B. , Self, D.W. , Iadarola, M.J. , Nakabeppu, Y. , Duman, R.S. & Nestler, E.J. (1994) Induction of a long‐lasting AP‐1 complex composed of altered Fos‐like proteins in brain by chronic cocaine and other chronic treatments. Neuron 13, 1235–1244. 7946359 10.1016/0896-6273(94)90061-2

[gbb12422-bib-0015] Itoh, Y. , Masuyama, N. , Nakayama, K. , Nakayama, K.I. & Gotoh, Y. (2007) The cyclin‐dependent kinase inhibitors p57 and p27 regulate neuronal migration in the developing mouse neocortex. J Biol Chem 282, 390–396. 17092932 10.1074/jbc.M609944200

[gbb12422-bib-0016] John, R.M. , Ainscough, J.F. , Barton, S.C. & Surani, M.A. (2001) Distant cis‐elements regulate imprinted expression of the mouse p57( Kip2) (Cdkn1c) gene: implications for the human disorder, Beckwith‐‐Wiedemann syndrome. Hum Mol Genet 10, 1601–1609. 11468278 10.1093/hmg/10.15.1601

[gbb12422-bib-0017] Joseph, B. , Wallen‐Mackenzie, A. , Benoit, G. , Murata, T. , Joodmardi, E. , Okret, S. & Perlmann, T. (2003) p57(Kip2) cooperates with Nurr1 in developing dopamine cells. Proc Natl Acad Sci USA 100, 15619–15624. 14671317 10.1073/pnas.2635658100 PMC307617

[gbb12422-bib-0018] Jupp, B. , Murray, J.E. , Jordan, E.R. , Xia, J. , Fluharty, M. , Shrestha, S. , Robbins, T.W. & Dalley, J.W. (2016) Social dominance in rats: effects on cocaine self‐administration, novelty reactivity and dopamine receptor binding and content in the striatum. Psychopharmacology 233, 579–589. 26554388 10.1007/s00213-015-4122-8 PMC4726718

[gbb12422-bib-0019] Lee, M.H. , Reynisdottir, I. & Massague, J. (1995) Cloning of p57KIP2, a cyclin‐dependent kinase inhibitor with unique domain structure and tissue distribution. Genes Dev 9, 639–649. 7729683 10.1101/gad.9.6.639

[gbb12422-bib-0020] Lindzey, G. , Winston, H. & Manosevitz, M. (1961) Social dominance in inbred mouse strains. Nature 191, 474–476. 13762409 10.1038/191474a0

[gbb12422-bib-0021] Matsuoka, S. , Edwards, M.C. , Bai, C. , Parker, S. , Zhang, P. , Baldini, A. , Harper, J.W. & Elledge, S.J. (1995) p57KIP2, a structurally distinct member of the p21CIP1 Cdk inhibitor family, is a candidate tumor suppressor gene. Genes Dev 9, 650–662. 7729684 10.1101/gad.9.6.650

[gbb12422-bib-0022] McGrath, J. & Solter, D. (1984) Completion of mouse embryogenesis requires both the maternal and paternal genomes. Cell 37, 179–183. 6722870 10.1016/0092-8674(84)90313-1

[gbb12422-bib-0023] McNamara, G.I. , Davis, B.A. , Dwyer, D.M. , John, R.M. & Isles, A.R. (2016) Behavioural abnormalities in a novel mouse model for silver Russell syndrome. Hum Mol Genet 25, 5407–5417. 27798108 10.1093/hmg/ddw357 PMC5418837

[gbb12422-bib-0024] Nestler, E.J. (2001) Molecular basis of long‐term plasticity underlying addiction. Nat Rev Neurosci 2, 119–128. 11252991 10.1038/35053570

[gbb12422-bib-0025] Ong, Z.Y. & Muhlhausler, B.S. (2011) Maternal "junk‐food" feeding of rat dams alters food choices and development of the mesolimbic reward pathway in the offspring. FASEB J 25, 2167–2179. 21427213 10.1096/fj.10-178392 PMC3114523

[gbb12422-bib-0026] Pena, C.J. , Neugut, Y.D. , Calarco, C.A. & Champagne, F.A. (2014) Effects of maternal care on the development of midbrain dopamine pathways and reward‐directed behavior in female offspring. Eur J Neurosci 39, 946–956. 24446918 10.1111/ejn.12479

[gbb12422-bib-0027] Radford, E.J. , Isganaitis, E. , Jimenez‐Chillaron, J. , Schroeder, J. , Molla, M. , Andrews, S. , Didier, N. , Charalambous, M. , McEwen, K. , Marazzi, G. , Sassoon, D. , Patti, M.E. & Ferguson‐Smith, A.C. (2012) An unbiased assessment of the role of imprinted genes in an intergenerational model of developmental programming. PLoS Genet 8, e1002605. 22511876 10.1371/journal.pgen.1002605 PMC3325178

[gbb12422-bib-0028] Russo, S.J. & Nestler, E.J. (2013) The brain reward circuitry in mood disorders. Nat Rev Neurosci 14, 609–625. 23942470 10.1038/nrn3381 PMC3867253

[gbb12422-bib-0029] Smith, F.M. , Garfield, A.S. & Ward, A. (2006) Regulation of growth and metabolism by imprinted genes. Cytogenet Genome Res 113, 279–291. 16575191 10.1159/000090843

[gbb12422-bib-0030] Smith, K.S. , Berridge, K.C. & Aldridge, J.W. (2011) Disentangling pleasure from incentive salience and learning signals in brain reward circuitry. Proc Natl Acad Sci USA 108, E255–E264. 21670308 10.1073/pnas.1101920108 PMC3131314

[gbb12422-bib-0031] Surani, M.A. , Barton, S.C. & Norris, M.L. (1984) Development of reconstituted mouse eggs suggests imprinting of the genome during gametogenesis. Nature 308, 548–550. 6709062 10.1038/308548a0

[gbb12422-bib-0032] Tellez, L.A. , Han, W. , Zhang, X. , Ferreira, T.L. , Perez, I.O. , Shammah‐Lagnado, S.J. , van den Pol, A.N. & de Araujo, I.E. (2016) Separate circuitries encode the hedonic and nutritional values of sugar. Nat Neurosci 19, 465–470. 26807950 10.1038/nn.4224 PMC4767614

[gbb12422-bib-0033] Tunster, S.J. , Jensen, A.B. & John, R.M. (2013) Imprinted genes in mouse placental development and the regulation of fetal energy stores. Reproduction 145, R117–R137. 23445556 10.1530/REP-12-0511

[gbb12422-bib-0034] Tury, A. , Mairet‐Coello, G. & DiCicco‐Bloom, E. (2011) The cyclin‐dependent kinase inhibitor p57Kip2 regulates cell cycle exit, differentiation, and migration of embryonic cerebral cortical precursors. Cereb Cortex 21, 1840–1856. 21245411 10.1093/cercor/bhq254 PMC3138513

[gbb12422-bib-0035] Usiello, A. , Baik, J.H. , Rouge‐Pont, F. , Picetti, R. , Dierich, A. , LeMeur, M. , Piazza, P.V. & Borrelli, E. (2000) Distinct functions of the two isoforms of dopamine D2 receptors. Nature 408, 199–203. 11089973 10.1038/35041572

[gbb12422-bib-0036] Van de Pette, M. , Abbas, A. , Feytout, A. , McNamara, G. , Bruno, L. , To, W.K. , Dimond, A. , Sardini, A. , Webster, Z. , McGinty, J. , Paul, E.J. , Ungless, M.A. , French, P.M. , Withers, D.J. , Uren, A. , Ferguson‐Smith, A.C. , Merkenschlager, M. , John, R.M. & Fisher, A.G. (2017) Visualizing changes in Cdkn1c expression links early‐life adversity to imprint Mis‐regulation in adults. Cell Rep 18, 1090–1099. 28147266 10.1016/j.celrep.2017.01.010 PMC5300902

[gbb12422-bib-0037] Vucetic, Z. , Kimmel, J. , Totoki, K. , Hollenbeck, E. & Reyes, T.M. (2010a) Maternal high‐fat diet alters methylation and gene expression of dopamine and opioid‐related genes. Endocrinology 151, 4756–4764. 20685869 10.1210/en.2010-0505 PMC2946145

[gbb12422-bib-0038] Vucetic, Z. , Totoki, K. , Schoch, H. , Whitaker, K.W. , Hill‐Smith, T. , Lucki, I. & Reyes, T.M. (2010b) Early life protein restriction alters dopamine circuitry. Neuroscience 168, 359–370. 20394806 10.1016/j.neuroscience.2010.04.010 PMC2873068

[gbb12422-bib-0039] Wang, F. , Zhu, J. , Zhu, H. , Zhang, Q. , Lin, Z. & Hu, H. (2011) Bidirectional control of social hierarchy by synaptic efficacy in medial prefrontal cortex. Science 334, 693–697. 21960531 10.1126/science.1209951

[gbb12422-bib-0040] Westbury, J. , Watkins, M. , Ferguson‐Smith, A.C. & Smith, J. (2001) Dynamic temporal and spatial regulation of the cdk inhibitor p57(kip2) during embryo morphogenesis. Mech Dev 109, 83–89. 11677056 10.1016/s0925-4773(01)00512-3

[gbb12422-bib-0041] Wise, R.A. (2013) Dual roles of dopamine in food and drug seeking: the drive‐reward paradox. Biol Psychiatry 73, 819–826. 23044182 10.1016/j.biopsych.2012.09.001 PMC3548035

[gbb12422-bib-0042] Yan, Y. , Frisen, J. , Lee, M.H. , Massague, J. & Barbacid, M. (1997) Ablation of the CDK inhibitor p57Kip2 results in increased apoptosis and delayed differentiation during mouse development. Genes Dev 11, 973–983. 9136926 10.1101/gad.11.8.973

[gbb12422-bib-0043] Ye, W. , Mairet‐Coello, G. , Pasoreck, E. & Dicicco‐Bloom, E. (2009) Patterns of p57Kip2 expression in embryonic rat brain suggest roles in progenitor cell cycle exit and neuronal differentiation. Dev Neurobiol 69, 1–21. 18814313 10.1002/dneu.20680 PMC2967216

[gbb12422-bib-0044] Zhang, P. , Liegeois, N.J. , Wong, C. , Finegold, M. , Hou, H. , Thompson, J.C. , Silverman, A. , Harper, J.W. , DePinho, R.A. & Elledge, S.J. (1997) Altered cell differentiation and proliferation in mice lacking p57KIP2 indicates a role in Beckwith‐Wiedemann syndrome. Nature 387, 151–158. 9144284 10.1038/387151a0

