# Supplementary material for: Dopaminergic and behavioural changes in a loss‐of‐imprinting model of Cdkn1c
Source: Genes Brain Behav. 2017 Sep 15;17(2):149–57. doi: 10.1111/gbb.12422 (PMC5836939; doi:10.1111/gbb.12422)
Supplement: Supplementary file 1 — Figure S1: Cdkn1c BACx1 animals had elevated neural Cdkn1c expression at e18.5 as determined by qPCR. Data shown ±SEM. **P < 0.01. Figure S2: There was no effect of genotype on dopaminergic state in the striatum of Cdkn1c BACLacZ animals and WT littermates. (a) Adult Th expression normalized to WT (b) Dat expression normalized to WT. (c) Whole tissue dopamine concentration. (d) Th immuno‐reactivity in the striatum (e). Average NeuN+ve cell count. Dorsal striatum (DS) ventral striatum (VS) Data shown ±SEM. Figure S3: There was no effect of genotype on motivation to obtain a sucrose reward compared between Cdkn1c BACLacZ animals and WT littermates. (a) Average number of trials completed during CRF trials (b) BP when the number of nose‐pokes required to obtain a reward increased within a session. (c) Duration of time between successive nose‐pokes (d) Average time to complete a trial. Data shown ±SEM. [file GBB-17-149-s001.docx]

**Dopaminergic and behavioral changes in a loss-of-imprinting model of *Cdkn1c***

**– SUPPLEMENTARY MATERIALS**

Gráinne I. McNamara^1^, Brittany A. Davis^2^, Molly Browne^2^, Trevor Humby^1,3^, Jeffrey W. Dalley^4,5^, Jing Xia^4^, Rosalind M. John^2^ & Anthony R. Isles^1*^

^1^Behavioural Genetics Group, MRC Centre for Neuropsychiatric Genetics and Genomics, Neuroscience and Mental Health Research Institute, Cardiff University, Cardiff, United Kingdom CF24 4HQ

^2^ School of Biosciences, Cardiff University, Cardiff, United Kingdom, CF10 3AX

^3^ School of Psychology, Cardiff University, Cardiff, United Kingdom CF10 3AT

^4^Departement of Psychology, University of Cambridge, Cambridge, United Kingdom, CB2 3RQ

^5^Department of Psychiatry, University of Cambridge, Cambridge, United Kingdom, CB2 0SZ

*Corresponding author: Email: IslesAR1@cardiff.ac.uk; Tel. +44(0)2920 688467

# RESULTS

## Basal dopaminergic state was not altered in *Cdkn1c*^BACLacZ^ control mice

In adult *Cdkn1c*^BACLacZ^ control mice mRNA levels in the striatum of the gene *Th* were unaltered relative to WT littermate (Figure S1A; main effect of GENOTYPE: F_1,5_=2.2, p=0.2). Similarly, expression of *Dopamine transporter* (*Dat*) mRNA was also equivalent (Figure S1B; t(8)=-0.1, p=0.923) in the dorsal striatum of *Cdkn1c*^BACLacZ^ animals compared with WT littermates. This lack of a difference in mRNA levels of key dopaminergic genes was supported by equivalence between *Cdkn1c*^BACLacZ^ animals and WT littermates in whole tissue dopamine concentration in the dorsal striatum (Figure S1C; main effect of genotype F_1,14_=0.481, p=0.499), and Th protein immunoreactivity in the dorsal and ventral striatum of (Figure S1D; main effect of GENOTYPE F_1,6_=0.617, p=0.662). Finally, there was no difference between *Cdkn1c*^BACLacZ^ animals and WT littermates in relative cell counts in the striatum (F_1,7_=0.025, p=0.879) or surrounding cortex (F_1,7_=0.732, p=0.425), as determined by number of NeuN positive cells (Figure S1E).

## Motivation in a PR task was not altered in *Cdkn1c*^BACLacZ^ control mice

During CRF trials, where a single nose-poke elicited an 8% sucrose reward, there was no difference in number of trials completed between WT and *Cdkn1c*^BACLacZ^ animals (Figure S2A; main effect of GENOTYPE: F_1,23_=0.083, p=0.776). In a progressive ratio schedule, during which the number of nose pokes required to receive a reward ascends within a session *Cdkn1c*^BACLacZ^ animals also had an equivalent breakpoint to their WT littermates (Figure S2B; F_1,23_=1.012, p=0.33). The lack of difference in the main measure of motivation on this task was also reflected in additional measures, such as the inter nose-poke interval (Figure S2C; F_1,23_=0.236, p=0.61), and time to complete trials (Figure S2D; F_1,23_=0.036, p=0.85), in which *Cdkn1c*^BACLacZ^ animals performed at an equivalent level to their WT littermates.

**Figure S1**

| **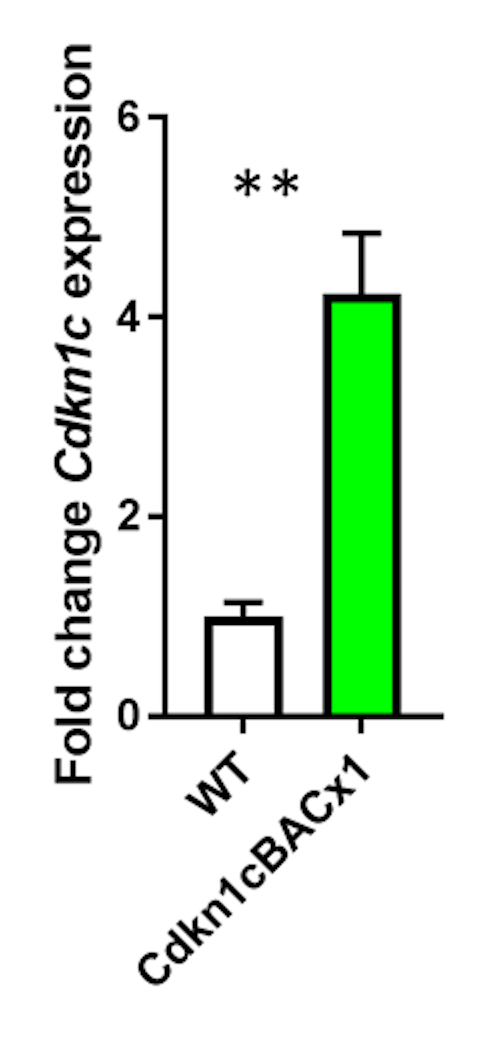** | Figure S1: *Cdkn1c*^BACx1^ animals had elevated neural *Cdkn1c* expression at e18.5 as determined by qPCR. Data shown ±SEM **p<0.01 |
| --- | --- |

**Figure S2**

| 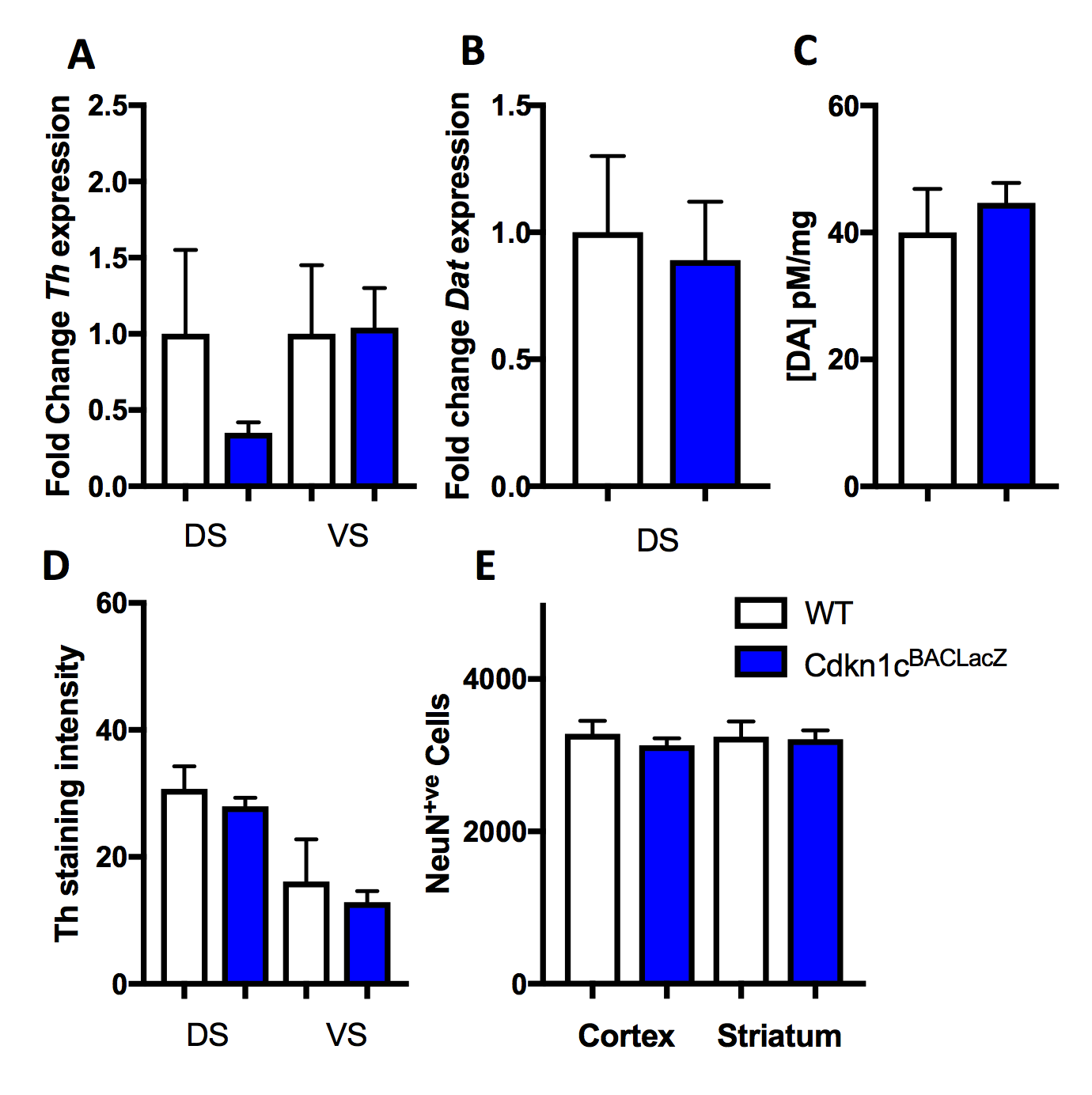 |
| --- |
| **Figure S2: There was no effect of genotype on dopaminergic state in the striatum of *Cdkn1c*^BACLacZ^ animals and WT littermates.** (A) Adult *Th* expression normalised to WT (B) *Dat* expression normalised to WT. (C) Whole tissue dopamine concentration. (D) Th immuno-reactivity in the striatum (E). Average NeuN^+ve^ cell count. Dorsal striatum (DS) ventral striatum (VS) Data shown ±SEM. |

**Figure S3**

| 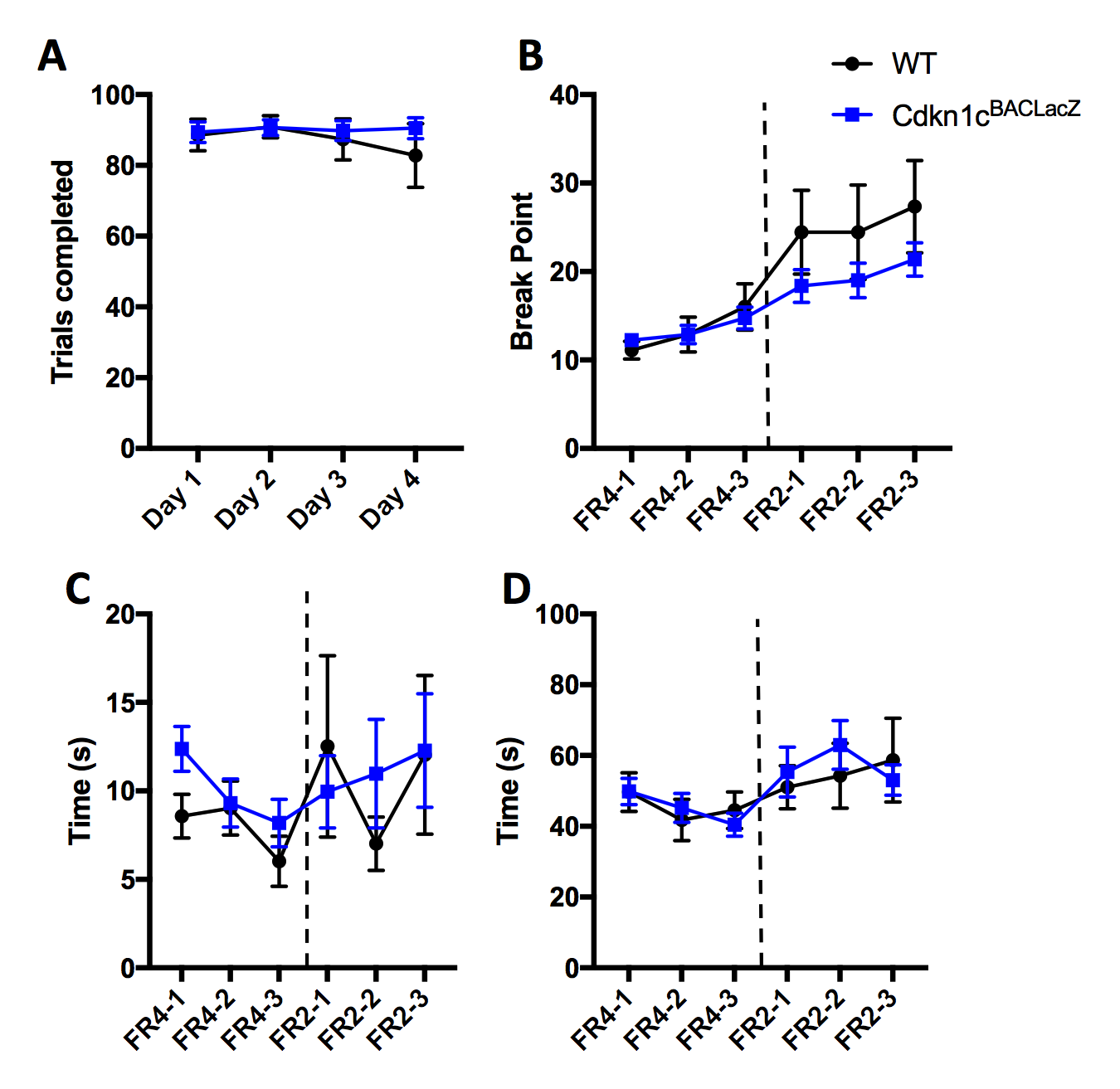 |
| --- |
| **Figure S3: There was no effect of genotype on motivation to obtain a sucrose reward compared between *Cdkn1c*^BACLacZ^ animals and WT littermates.** (A) Average number of trials completed during CRF trials (B) BP when the number of nose-pokes required to obtain a reward increased within a session. (C) Duration of time between successive nose-pokes (D) Average time to complete a trial. Data shown ±SEM. |
